# The pro-metastatic effect and mechanism of immune cells in tumor metastasis

**DOI:** 10.3389/fonc.2026.2007187

**Published:** 2026-10-29

**Authors:** Shitao Geng, Haihui Liang, Kun Lu, Changling Deng, Deqiang Bao, Hui Xu

**Affiliations:** 1 Department of Oncology, First Naval Hospital of Southern Theater Command, Zhanjiang, China; 2 Department of Neurosurgery, First Naval Hospital of Southern Theater Command, Zhanjiang, China

**Keywords:** immune cell, immune escape, immunosuppression, tumor metastasis, tumor microenvironment

## Abstract

Tumor metastasis is the leading cause of cancer-related deaths, and its occurrence is not only determined by the invasive capacity of tumor cells themselves but is also profoundly influenced by the dynamic remodeling of the immune system. Recent studies over the past decade have shown that the immune mechanisms in tumor metastasis have expanded from the classical failure of immune surveillance to a systemic process involving immune cell functional remodeling, the construction of the pre-metastatic niche (PMN), and tumor immune editing. This review systematically reviews the tumor promoting mechanisms of various immune cells such as T cells, tumor-associated macrophages (TAMs), myeloid-derived suppressor cells (MDSCs), tumor-associated neutrophils (TANs), regulatory B cells (Bregs), tumor-associated natural killer cells (TANKs) and dendritic cells (DCs) in each stage of metastasis, and critically discusses the limitations, conceptual controversies, and future directions of current research. This review seeks to establish a systematic framework to enhance the understanding of immune cell mechanisms involved in tumor metastasis.

## 1 Introduction

Tumor metastasis is the main cause of death in malignant tumors, and its occurrence is a complex biological process involving multiple steps and links, involving a series of cascading events such as tumor cell detachment from the primary lesion, local invasion, vascular infiltration, circulatory system survival, distant organ arrest, extravasation, and ultimately colonization and growth (invasion metastasis cascade) (1). In recent years, with the rapid development of technologies such as single-cell sequencing, spatial transcriptomics, and high-resolution *in vivo* imaging, the academic community’s understanding of tumor metastasis has shifted from the “tumor cell centric theory” to the “tumor microenvironment (TME) ecosystem theory”. In this ecosystem, immune cells become one of the core factors regulating the transfer process due to their high plasticity and functional diversity (2).

According to classical immunological theory, the immune system clears abnormal cells and inhibits metastasis through the mechanism of “cancer immune surveillance”. However, a large body of evidence suggests that immune cells, as the most plastic component in the TME, are no longer limited to clearing tumor cells. On the contrary, multiple immune cells acquire a pro metastatic phenotype under the regulation of tumor derived cytokines, chemokines, and metabolites, actively participating in the progression of various stages of metastasis (3). Most cancer-related deaths are attributed to metastasis, and the response rate of advanced metastatic tumors to existing treatment methods (including immune checkpoint blockade) is generally limited (4, 5). The latest research suggests that the immune microenvironment characteristics of metastatic tumors, rather than the primary tumor, are the key factors determining the efficacy of immunotherapy (6).

However, there are several issues worth examining in the current field. Firstly, most studies rely on animal models, and there are significant differences in immune cell function between humans and mice, which poses challenges for clinical translation. Secondly, the plasticity of immune cells allows the same cell type to perform completely opposite functions at different stages of metastasis, and a simple pro-tumor/anti-tumor binary is no longer sufficient to describe its complexity. Thirdly, the heterogeneity of the immune microenvironment in different metastatic sites further increases the difficulty of understanding the mechanism. This paper reviews relevant literature to explore the roles of various immune cells in tumor metastasis and the mechanisms by which they promote metastasis.

## 2 Methods

This review employs a narrative review design. We searched PubMed, Web of Science, and Scopus databases for literature published between January 2010 and August 2026, using combinations of the following search terms: “tumor metastasis, “ “immune microenvironment, “ “immunosuppression, “ “T cell exhaustion, “ “regulatory T cell, “ “tumor-associated macrophage, “ “myeloid-derived suppressor cell, “ “tumor-associated neutrophil, “ “regulatory B cell, “ “NK cell, “ “pre-metastatic niche, “ and “immune checkpoint blockade.” The above search strategy was used to identify major research advances in recent years; however, this study did not undergo the pre-registration or dual-blind screening process required for a systematic review and therefore does not meet the criteria for a systematic review.

## 3 The tumor promoting effects of multiple immune cells

### 3.1 T cells

#### 3.1.1 T cell dysfunction promotes metastasis

T cells, especially CD8 ^+^ cytotoxic T cells, are the backbone of adaptive anti-tumor immunity. However, during tumor progression, T cells often become dysfunctional due to multiple inhibitory mechanisms, manifested in three main forms: exhaustion, aging, and metabolic restriction. The depletion of T cells is characterized by a sustained decline in effector function and upregulation of inhibitory receptors expression; T cell aging involves telomere shortening and epigenetic changes, leading to loss of proliferative capacity; Metabolic limitation arises from nutritional competition and acidosis in the TME, which weakens the survival and function of T cells (7).

Research has shown that depletion of CD8 ^+^ T cells is an important mechanism for promoting metastasis. Exhausted T cells overexpress inhibitory receptors such as Programmed Cell Death Protein 1 (PD-1), Cytotoxic T-Lymphocyte-Associated Protein 4 (CTLA-4), and T-cell Immunoglobulin and Mucin-domain containing molecule 3 (TIM-3), accompanied by decreased cytokine secretion ability, making it difficult to effectively clear disseminated tumor cells (8, 9). Within metastatic lesions, these exhausted T cells frequently accumulate in an immunosuppressive microenvironment and are further inhibited by myeloid-derived suppressor cells (MDSCs) and regulatory T cells (Tregs), thereby hindering the effective clearance of disseminated tumor cells and promoting metastasis (10, 11). In addition, metabolic stress in the microenvironment of metastatic foci, including hypoxia, glucose deficiency, lactate accumulation, and oxidative lipid accumulation, can impair T cell mitochondrial function and induce metabolic reprogramming (12, 13). Meanwhile, tumor cells can even transport abnormal mitochondria containing high levels of reactive oxygen species to T cells through tunnel nanotubes or extracellular vesicles, accelerating T cell aging and functional loss (12). The elucidation of this mechanism offers a novel perspective on the exhaustion of T cell function during the circulatory phase and suggests that targeting mitochondrial transfer could be a promising strategy to enhance the effectiveness of immunotherapy.

T cell senescence is commonly present in malignant tumors and affects multiple stages of metastasis (14, 15). The infiltration ability of aging T cells into tumors decreases, and even if they enter the tumor, their cytotoxic function is significantly weakened (10, 16). Concurrently, senescent T cells secrete senescence-associated secretory phenotype (SASP) factors, including various inflammatory cytokines and chemokines, which facilitate the recruitment of inhibitory immune cells such as MDSCs and Tregs. This process further exacerbates the local immune suppression within the tumor, creating favorable conditions for tumor metastasis (10). For tumor cells to metastasize to distant sites, they must withstand anoikis and blood shear stress, while also avoiding immune system attacks. Aging T cells have a reduced supervisory function during this process, facilitating the survival of circulating tumor cells in the bloodstream and their arrival at distant organs. Studies indicate that senescent T cells are also crucial in forming the pre-metastatic niche (PMN) (10). The dysfunction of senescent T cells impairs their ability to effectively recognize and eliminate disseminated tumor cells during the early stages of metastasis, thereby facilitating the formation of micrometastases.

#### 3.1.2 Regulatory T cells: the hub of immune suppression

Tregs constitute a crucial subpopulation of cells responsible for maintaining immune tolerance. However, within the TME, the accumulation of Tregs is frequently correlated with poor prognosis. Competitive depletion of interleukin (IL) -2 by Tregs is a well-established mechanism in the field of tumor immunity. Research conducted by Kahn et al. (17) has further demonstrated that tumor lymph node metastasis is associated with the role of Tregs in tumor-draining lymph nodes. Tregs in tumor-draining lymph nodes highly express the IL-2 receptor, competitively depleting IL-2 in the microenvironment. This prevents CD8^+^ T cells, which should normally be activated from receiving sufficient IL-2 signaling, thereby rendering them functionally inactivated. As a result, they express almost no key effector molecules such as granzyme-B and perforin, and are unable to effectively kill cancer cells that have invaded the lymph nodes. Consequently, lymph nodes, which should serve as “immune outposts”, become a vulnerable point for tumor metastasis, allowing cancer cells that have metastasized to the lymph nodes to survive and proliferate more easily, ultimately establishing metastatic foci (17). Tregs suppress anti-tumor immune responses through multiple mechanisms, including the secretion of inhibitory cytokines such as IL-10 and transforming growth factor-beta (TGF-β); inhibition of T cell co-stimulatory signals via the interaction between CTLA-4 and antigen-presenting cells; and direct cytotoxic effects on T cells or dendritic cells (DCs) (13, 18). During the initial stage of metastasis, TGF - β secreted by Tregs is a key factor in inducing epithelial mesenchymal transition (EMT), upregulating transcription factors such as SNAIL, TWIST, ZEB1, and promoting tumor cell migration and invasion ability. Tregs collaborate with tumor associated macrophages (TAMs) and MDSCs to construct immunosuppressive TMEs, creating conditions for tumor cell invasion (3, 11).

#### 3.1.3 Regulation of microenvironment during distant colonization stage

Prior to the dissemination of tumor cells to distant organs, the primary tumor lesion can influence the microenvironment of the target organ by secreting soluble factors and exosomes, thereby establishing a PMN that facilitates tumor colonization. T cells exhibit a dual role in this context. On one hand, PMNs frequently accumulate immunosuppressive cells, such as Tregs and MDSCs, which create an immunosuppressive microenvironment conducive to the colonization of disseminated tumor cells. Tumor-derived factors, including TGF-β and vascular endothelial growth factor (VEGF), can promote the differentiation of T cells in target organs into Tregs, thereby suppressing local anti-tumor immune responses (11). Conversely, pre-existing tissue-resident memory T (TRM) may exert a protective function by initiating local immune responses upon the arrival of tumor cells, effectively eliminating disseminated tumor cells and preventing metastatic formation (13).

Upon colonizing the target organ, disseminated tumor cells engage in continuous interactions with T cells within the local microenvironment, undergoing a series of immune editing processes. These processes are categorized into three distinct stages: elimination, equilibrium, and escape. During the elimination phase, locally activated CTLs effectively eradicate disseminated tumor cells by recognizing tumor-specific antigens. Nevertheless, some tumor cells may acquire immunogenic alterations, allowing them to evade immune detection and transition into a state of equilibrium. In this equilibrium phase, a dynamic standoff ensues between tumor cells and T cells, wherein tumor cells remain dormant while T cells are unable to completely eliminate them (13). This stage may persist for several months or even years, often manifesting clinically as occult micrometastasis. Eventually, tumor cells overcome immune surveillance through mechanisms such as antigen loss, downregulation of major histocompatibility complex (MHC) molecules, or the induction of an immunosuppressive microenvironment, thereby entering the escape phase and culminating in the formation of clinically detectable metastases (13).

While existing studies have elucidated potential mechanisms by which T cells influence tumor metastasis, the heterogeneity of immune microenvironments across various metastatic sites complicates the comprehensive understanding of T cell functions. T cell activity within the metastatic microenvironment can either inhibit or promote tumor growth, contingent upon specific contextual factors. Furthermore, the intricate interactions among T cells, host factors, and other tumor components necessitate additional research to thoroughly elucidate their roles within the metastatic cascade.

### 3.2 TAMs

TAMs are pivotal cells that produce cytokines, chemokines, and growth factors, which stimulate T cells to release inhibitory immune checkpoint proteins, thereby establishing an immunosuppressive TME (19). These macrophages are activated by various signals within the TME and exert significant influence on tumor initiation, progression, metastasis, immune modulation, and angiogenesis (20). These processes are not isolated; rather, they constitute a continuous and mutually reinforcing vicious cycle. Initially, the primary tumor recruits circulating monocytes into the tumor milieu by secreting factors such as C-C motif chemokine ligand 2 (CCL2) (12) or remotely influences distant organs via exosomes (21). Subsequently, signals such as IL-4 and granulocyte-macrophage colony-stimulating factor (GM-CSF) present in the TME polarize these monocytes into TAMs exhibiting M2-like characteristics (9). Under the influence of the primary tumor, TAMs enhance PD-L1 expression and secrete lactate in organs including the lungs and liver by activating aryl hydrocarbon receptor (AHR) signaling pathways (9, 21). This process inhibits local immune responses and establishes an immunosuppressive pre-metastatic microenvironment, thereby priming these sites for the arrival of tumor cells. Upon the arrival of circulating tumor cells, the pre-existing TAMs facilitate their survival, colonization, and subsequent formation of metastatic lesions by maintaining immune suppression and providing growth factors such as epidermal growth factor (EGF) and VEGF (22).

#### 3.2.1 Modulation tumor cell characteristics and metastatic potential

TAMs significantly influence the biological characteristics of tumor cells and enhance their metastatic potential through a variety of mechanisms. A critical process in the acquisition of migratory and invasive capabilities by tumor cells is the EMT, for which TAMs serve as pivotal inducers. Empirical studies have demonstrated that TAMs facilitate the activation of EMT-related signaling pathways in tumor cells by secreting a range of signaling molecules, including TGF-β, Wnt, and IL-6. This secretion promotes the downregulation of epithelial markers, such as E-cadherin, and the upregulation of mesenchymal markers, such as N-cadherin and vimentin, thereby augmenting the motility and invasiveness of the tumor cells (23, 24). In addition, the study found that long non-coding RNAs (lncRNAs) derived from M2-type TAMs (such as H19, AFAP1-AS1, and CRNDE) can induce EMT in tumor cells and stimulate tumor proliferation and metastasis through a series of cascading reactions (25).

In addition to secreting soluble factors, TAMs can influence tumor cells through direct intercellular contact. Studies have demonstrated that TAMs can transmit signals to tumor cells via membrane-bound ligand-receptor interactions, such as epidermal growth factor receptor (EGFR)/Prostaglandin E2 (PGE2) and CD80/CTLA-4, thereby enhancing the stem cell-like properties and drug resistance of tumor cells (24). In a colorectal cancer liver metastasis model, Kupffer cells, which are liver-resident macrophages located in the hepatic sinusoids, can directly interact with circulating colorectal cancer cells. This interaction facilitates the capture and settlement of these cancer cells, thereby promoting liver metastasis (12). Remarkably, TAMs may also undergo cell fusion with tumor cells, resulting in hybrid cells that exhibit both the motility of macrophages and the unlimited proliferative capacity of tumor cells. These hybrid cells may play a unique role in tumor metastasis (25). Furthermore, the interplay between TAMs and circulating tumor cells (CTCs) has emerged as a focal point of research in recent years. Studies have demonstrated that TAMs facilitate the resistance of CTCs to fluid shear stress and anoikis within the bloodstream. This is achieved through mechanisms including platelet activation and fibrin deposition, thereby enhancing the survival rate of CTCs and augmenting metastatic efficiency (26).

#### 3.2.2 Regulation extracellular matrix remodeling

The remodeling of the extracellular matrix (ECM) is a critical process in the invasion and metastasis of tumor cells. TAMs significantly influence the physical and chemical characteristics of the ECM by secreting a variety of proteases and modulating matrix cross-linking, thereby establishing conditions conducive to tumor metastasis.

TAMs serve as critical sources of various matrix-degrading enzymes, including Matrix Metalloproteinases (MMPs), proteases, and serine proteases such as urokinase-type plasminogen activator (uPA). These enzymes facilitate the degradation of multiple ECM components, including collagen, fibronectin, and laminin, thereby compromising the integrity of the basement membrane and creating pathways conducive to tumor cell migration (27, 28). Empirical evidence indicates that in breast and colon cancers, TAMs exhibit overexpression of MMP-2, MMP-9, and MMP-14, which correlates positively with increased tumor invasion depth and metastasis (29, 30). In addition to facilitating the degradation of the ECM, TAMs can modify its physical characteristics by promoting collagen cross-linking and fibrosis. TAMs achieve this by activating cancer-associated fibroblasts (CAFs) through the secretion of transforming growth TGF-β and PDGF, thereby stimulating the production of substantial quantities of ECM components. Concurrently, TAMs express cross-linking enzymes such as lysyl oxidase (LOX), which enhance the rigidity and tension of the matrix. This fibrotic microenvironment not only augments the migratory capacity of tumor cells but also influences the recruitment and polarization of macrophages by activating integrin signaling pathways, thereby facilitating tumor progression (26, 31–33).

#### 3.2.3 Promotion angiogenesis and lymphangiogenesis

The tumor vasculature serves as a conduit for hematogenous metastasis of tumor cells, with TAMs playing a crucial role in promoting tumor angiogenesis. TAMs facilitate endothelial cell proliferation and lumen formation by secreting a variety of angiogenic factors, including VEGF, platelet-derived growth factor (PDGF), basic fibroblast growth factor (bFGF), and IL-8 (12, 26). Studies have demonstrated a significant correlation between the distribution of TAMs within tumors and vascular density, particularly in hypoxic regions. In response to hypoxic conditions, TAMs enhance angiogenesis by upregulating HIF-1α and VEGF expression. Furthermore, TAMs contribute to angiogenesis by secreting various MMPs, which degrade the ECM, thereby creating space for new blood vessel formation and releasing growth factors sequestered within the matrix (26, 34, 35). It is noteworthy that TAMs facilitate the formation of tumor vasculature characterized by abnormal structures and impaired functions, which paradoxically supports tumor metastasis (34, 36). The increased permeability of immature blood vessels facilitates the entry of tumor cells into the vascular lumen. Concurrently, vascular dysfunction induces hypoxic conditions within the tumor microenvironment, thereby activating HIF-1α signaling. This activation establishes a positive feedback loop that enhances angiogenesis and promotes tumor invasion (37).

Lymphatic angiogenesis constitutes a critical pathway for tumor cell metastasis via the lymphatic system, with TAMs playing a significant role in modulating this process. TAMs facilitate lymphangiogenesis and tumor metastasis through multiple mechanisms, including direct participation in lymphangiogenesis and the regulation of related signaling pathways via secreted factors. Specifically, CCL22 expressed in TAMs modulates the expression of VEGF-C through the IL-4/STAT6 signaling pathway, subsequently activating VEGFR-3 receptors on lymphatic endothelial cells, thereby promoting lymphangiogenesis and lymph node metastasis in patients with tongue squamous cell carcinoma (12, 38). Studies have demonstrated that TAMs not only enhance lymphatic vessel expansion by secreting soluble factors but also directly engage in lymphangiogenesis through distinct cellular subpopulations, such as myeloid-derived lymphoid endothelial progenitor cells (M-LECP) (39). Elucidating these mechanisms not only advances our understanding of the intricate process of tumor metastasis but also offers novel insights for developing therapeutic strategies targeting TAMs.

#### 3.2.4 Shape the immunosuppressive microenvironment

TAMs play a pivotal role in modulating the immunosuppressive microenvironment through multiple mechanisms. Firstly, TAMs directly suppress T cell activation and proliferation by secreting immunosuppressive cytokines, including TGF-β, IL-10, and PGE2. Additionally, TAMs exhibit high expression levels of immune checkpoint molecules such as PD-L1 and PD-L2, which interact with PD-1 receptors on T cells, leading to T cell dysfunction (12, 40). Furthermore, research has demonstrated that TAMs can recruit Tregs by secreting the chemokine CCL22, thereby amplifying immune suppression (24). Another critical mechanism by which TAMs inhibit anti-tumor immunity is through the impairment of antigen presentation function. A recent study has demonstrated that Parkin molecules in TAMs facilitate the autophagic degradation of MHC-I molecules via the AMPK-Parkin-ATG5 signaling pathway. This process diminishes the antigen presentation capabilities of TAMs, impairing the ability of CD8+ T cells to effectively recognize and eliminate tumor cells (12). In animal models, TAMs with Parkin knockout exhibit enhanced antigen presentation and T cell activation, leading to a significant inhibition of tumor growth. When combined with PD-L1 antibody treatment, this approach yields synergistic anti-tumor effects (12). Furthermore, TAMs contribute to immune suppression through metabolic reprogramming. For instance, TAMs exhibit high expression levels of indoleamine 2, 3-dioxygenase (IDO) and arginase-1 (Arg-1), which catalyze the catabolism of tryptophan and arginine, respectively. This metabolic activity depletes essential amino acids necessary for T cell activation and generates toxic metabolites that inhibit T cell function (41).

#### 3.2.5 Mediate the formation of the PMN

The PMN denotes a sequence of molecular and cellular alterations in distant organs that precede the arrival of tumor cells, thereby creating a conducive “soil” for tumor cell colonization and proliferation. TAMs are pivotal in the establishment of this process. Empirical studies have demonstrated that primary tumors can recruit bone marrow-derived cells to distant organs through the secretion of exosomes, cytokines, and soluble factors, thereby establishing a pre-metastatic microenvironment (12, 39). Among these bone marrow-derived cells, monocytes and macrophages constitute the primary compone. In the pre-metastatic microenvironment, TAMs facilitate metastasis through several mechanisms: (1) Immunosuppression: TAMs recruit MDSCs and Tregs, thereby suppressing local anti-tumor immunity; (2) Inflammatory milieu: TAMs secrete pro-inflammatory factors, including tumor necrosis factor-alpha (TNF-α), IL-1β, and S100A8/A9, fostering a chronic inflammatory environment that enhances tumor cell homing and colonization; (3) Vascular permeability: By releasing factors like VEGF and angiopoietin-2 (ANGPT2), TAMs increase vascular permeability, thereby promoting tumor cell extravasation (39, 41, 42).

The pre-metastatic microenvironment of various organs exhibits organ-specific characteristics, with TAMs playing a crucial role in these specificities. For instance, in brain metastasis, both microglia (resident macrophages of the brain) and peripherally derived macrophages collaboratively contribute to the establishment of the pre-metastatic microenvironment (39). In the context of bone metastasis, osteoclasts, a specialized form of macrophages, promote metastasis through the process of bone remodeling (43, 44). Additionally, the interaction between Kupffer cells and hepatic stellate cells constitutes a pivotal mechanism in liver metastasis (45). Understanding these organ-specific mechanisms is of paramount importance for the development of precise therapeutic strategies targeting specific metastatic sites.

It should be pointed out that TAMs exhibit high heterogeneity, often representing intermediate phenotypes between M1 and M2 states, and their functional characteristics are closely related to spatial distribution. Spatial analysis shows that there are TAMs subgroups within the same tumor that simultaneously express M1 and M2 genes, and the spatial distribution of M1 and M2 TAMs can more accurately reflect prognosis than simple phenotype classification. These findings emphasize the need to move beyond simplified M2 like classification and towards a spatially resolved, context dependent understanding of TAMs biology, in order to better understand the mechanism by which TAMs promote metastasis.

The high plasticity of TAMs is not only the root cause of their metastasis-promoting function but also provides a target for therapeutic intervention. The functional state of TAMs is not irreversible, reprogramming strategies to convert them from a tumor-promoting M2-like phenotype to an antitumor M1-like phenotype, or to restore their antigen-presenting and phagocytic functions, have emerged as highly promising approaches for anti-metastasis therapy. Current therapeutic strategies targeting TAMs primarily fall into three categories, inhibiting TAMs recruitment, eliminating specific TAMs subpopulations, and reprogramming TAMs polarization. Among these, reprogramming strategies are attracting increasing attention because they do not rely on the complete elimination of TAMs and can preserve other beneficial functions of macrophages (17). Recent studies have revealed a new mechanism regulating macrophage immune activation. Xia et al. (46) found that the antimicrobial peptide BSN-37 enhances macrophage immune activation through a circBptf-Rbm14-mediated regulatory axis, providing new insights into the use of innate immune regulatory molecules to restore macrophage antitumor function. At the same time, the key role of macrophage-derived lncRNAs in tumor immunoregulation is receiving increasing attention. Ramalingam et al. (25) conducted a systematic review of the functions of macrophage-derived lncRNAs in tumor immune evasion, microenvironment remodeling, and the regulation of treatment sensitivity, noting that these lncRNAs, as key immunoregulatory molecules, hold promise as innovative targets for personalized immunotherapy. These findings suggest that, in addition to traditional polarization and reprogramming strategies, targeting intrinsic regulatory networks within macrophages (e.g. epigenetic regulators and immune activation signaling pathways) may represent a new direction for restoring antitumor immunity in macrophages.

### 3.3 MDSCs

MDSCs are a class of immature myeloid cells that are typically classified into two major subsets: polymorphonuclear MDSCs (PMN-MDSCs) and mononuclear MDSCs (M-MDSCs), each with distinct phenotypic and functional characteristics. It should be noted that the relative abundance, migration preferences, and immunosuppressive activity of MDSC subpopulations are highly context- and cancer-type-dependent; observations may vary across different tumor types, disease stages, metastatic organs, and experimental models. In certain tumor models or specific cancer types, M-MDSCs may constitute the predominant population within the primary tumor and exhibit strong immunosuppressive activity, whereas PMN-MDSCs are relatively scarce in the primary tumor and are preferentially recruited to metastatic sites; however, this pattern is not universal across all tumors, and the distribution of these subpopulations and their functional contributions may vary depending on tumor type and microenvironmental context (13). Overall, MDSCs can promote tumor metastasis through mechanisms such as contributing to the establishment of an immunosuppressive microenvironment, facilitating PMN formation, angiogenesis, and tumor cell invasion (10).

#### 3.3.1 Building an immunosuppressive microenvironment

This is the most core and fundamental function of MDSCs. In order for tumor cells to successfully metastasize, they must first evade the surveillance and attack of the immune system, and MDSCs are the main force in creating this immune exemption environment.

Firstly, MDSCs have the capacity to directly inhibit cytotoxic T cells. Prior research has demonstrated that MDSCs exhibit high expression levels of Arg-1, which depletes arginine within the TME. Arginine is a critical amino acid necessary for the proliferation and activation of T cells; its depletion can lead to the downregulation of the CD3 zeta chain of the T cell receptor, thereby arresting the T cell proliferation cycle and impairing the T cells’ ability to combat tumor cells (47). Granulocytic MDSCs (G-MDSCs) are capable of producing substantial quantities of ROS and inducible nitric oxide synthase (iNOS), which interfere with T cell responses to signaling molecules, induce T cell apoptosis (48), and diminish Granzyme-B secretion (49). Moreover, molecules such as PD-L1, iNOS, and Arg-1, which are highly expressed in MDSCs, function not only as inhibitory agents but also as potent inducers of T cell depletion. The persistent antigenic stimulation within the immunosuppressive milieu orchestrated by MDSCs, coupled with the synergistic effect of these inhibitory signals, promotes the expression of a series of depletion-associated checkpoint molecules on T cells, including PD-1, TIM-3, and LAG-3. Consequently, T cells not only lose their functional capacity but also undergo phenotypic alterations. Even upon the removal of inhibitory signals, restoring these T cells to their normal functional state proves challenging. Furthermore, MDSCs can amplify this depletion process through the TGF-β1 signaling pathway (50).

MDSCs facilitate the induction and proliferation of Tregs with potent immunosuppressive capabilities through the secretion of cytokines, including TGF-β and IL-10. These Tregs subsequently suppress the activity of effector T cells, thereby establishing a synergistic inhibitory network with MDSCs (51). Tumor cells release specific chemokines, such as CXCL12, which attract MDSCs expressing the corresponding receptors, such as CXCR4. Upon arrival at the tumor site, the recruited MDSCs secrete factors like TGF-β1, which in turn stimulate tumor cells to produce additional CCL26. This CCL26 enhances the recruitment of MDSCs expressing CX3CR1, thereby forming a self-reinforcing positive feedback loop that intensifies the immunosuppressive milieu within the tumor, thus facilitating tumor cell evasion (50, 51).

#### 3.3.2 Promotion the survival of circulating tumor cells

The interaction between CTCs and MDSCs is recognized as a pivotal mechanism in cancer metastasis. Empirical evidence indicates that MDSCs not only facilitate tumor progression by suppressing the cytotoxic activity of T cells but also engage directly with CTCs to augment their metastatic potential (52). Specifically, PMN-MDSCs form heterogeneous aggregates with CTCs via ROS and Notch/Nodal signaling pathways, thereby enhancing the survival and dissemination of CTCs (15). Research has demonstrated that MDSCs and CTCs frequently coexist and can form cellular clusters within the peripheral blood of cancer patients. This physical association acts as a protective mechanism for CTCs, concealing their surface antigens and thereby reducing their vulnerability to detection and attack by circulating cytotoxic T lymphocytes or natural killer cells. Within these clusters, MDSCs may convey survival signals to CTCs through direct cell-to-cell contact or paracrine signaling, thereby inhibiting anoikis—a form of programmed cell death that occurs when cells detach from their native tissue environment—and significantly enhancing the survival rate of CTCs in the challenging conditions of the circulatory system. Upon reaching the vasculature of distant organs, the MDSC-CTCs clusters may facilitate CTC adhesion to the vascular endothelium through the secretion of specific factors, thereby aiding in their extravasation into the tissue and completing a critical step in the metastatic process (15, 53).

#### 3.3.3 Promotion the formation of PMN

In the PMN, MDSCs play a crucial role in facilitating tumor cell evasion and promoting tumor cell implantation. They achieve this by inhibiting immune cell activity, inducing matrix remodeling, and promoting angiogenesis (53). The primary tumor secretes various factors, including VEGF-A, TGF-β, and PGE2, to recruit MDSCs to specific distant organs. Upon arrival, MDSCs establish a localized immunosuppressive environment, thereby suppressing resident immune cells and removing barriers for the subsequent arrival of tumor cells. Concurrently, MDSCs secrete significant amounts of MMPs, such as MMP2 and MMP9 (54). These enzymes degrade the ECM and basement membrane, facilitating tumor cell embedding and releasing growth factors stored within the matrix. This process promotes angiogenesis, which supplies nutrients to metastatic lesions, and stimulates cell proliferation (23, 55). Additionally, proteins such as S100A8/A9 secreted by MDSCs can enhance inflammatory responses and increase vascular permeability, thereby facilitating the extravasation and settlement of tumor cells within tissue interstices (56).

### 3.4 Neutrophil

The inherent plasticity of neutrophils allows them to adapt to diverse cancer microenvironments, thereby exerting varying influences on cancer progression. According to previous research, neutrophils can be reprogrammed into a pro-tumorigenic state within the cancer microenvironment (57), contributing to vascular occlusion, tumor necrosis, and metastasis (13). Neutrophils are implicated in three critical stages of metastasis: within the primary tumor site, they facilitate tumor cell invasion by inducing EMT, remodeling the extracellular matrix, and promoting angiogenesis; in the circulatory system, they protect disseminated tumor cells by forming aggregates with CTCs, mediating immune evasion, and enhancing extravasation efficiency, and in distant organs, they establish conditions conducive to metastatic colonization by creating immunosuppressive PMN, altering the microenvironment, and reactivating dormant cells (13).

#### 3.4.1 Promotion tumor invasion

EMT is a pivotal mechanism that enables tumor cells to gain migratory and invasive capabilities. In the context of gastric cancer, tumor-associated neutrophils (TANs) secrete IL-17a, which activates the JAK2/STAT3 signaling pathway within tumor cells, thereby inducing EMT. Furthermore, TANs polarized towards the N2 phenotype suppress SLIT2 expression via miR-4745-5p and miR-3911, which are conveyed through exosomes, thus facilitating the metastasis of gastric cancer cells (58). Neutrophil elastase (NE) is integral to this process; upon entry into tumor cells, NE enhances the migration and aggregation of breast cancer cells by degrading insulin-like growth factor binding protein 2 (IGFBP-2). This degradation results in an increased concentration of free insulin-like growth factor 1 (IGF-1), which subsequently activates the IGF-1 receptor (IGF-1R) and its downstream Akt signaling pathway (58, 59). Furthermore, the ROS produced by neutrophils have the potential to cause DNA damage and genomic instability, thereby indirectly facilitating the progression of malignant phenotypes (13).

Neutrophils serve as a significant source of matrix metalloproteinases, particularly MMP-9. MMP-9 facilitates the degradation of basement membrane type IV collagen and laminin, thereby disrupting the tissue structural barrier and creating pathways for tumor cell invasion (58). Additionally, MMP-9 indirectly fosters angiogenesis by liberating VEGF bound to the matrix (16, 58). For instance, in bladder cancer cells, the transcription factor ETV4 enhances the secretion of CXCL1/8, thereby recruiting neutrophils that release VEGFA and MMP-9, which in turn promote lymphangiogenesis and lymph node metastasis (60). Beyond their role in protease activity, neutrophils contribute to matrix remodeling through the release of neutrophil extracellular traps (NETs). NETs, which are web-like structures composed of DNA scaffolds with citrullinated histones, NE, and myeloperoxidase, can ensnare tumor cells and degrade ECM components. In the context of hepatocellular carcinoma, the accumulation of acetyl CoA upregulates CXCL1 expression via histone H3 acetylation, thereby recruiting TANs and promoting NET formation, ultimately driving metastasis (58).

#### 3.4.2 Protection tumor cells in the circulatory system

Tumor cells that enter the bloodstream encounter numerous challenges, including shear stress, the loss of cellular clusters, apoptosis, and immune surveillance. An important adaptive strategy employed by tumor cells to overcome these challenges is the formation of aggregates with neutrophils. The literature indicates that CTCs and neutrophil aggregates possess a higher metastatic potential compared to individual CTCs, and their presence is associated with poor prognoses in breast cancer, gastric cancer, hepatocellular carcinoma, and other malignancies (16). The formation of CTC-neutrophil aggregates is governed by several molecular mechanisms: ① Chemokine axis: CTCs secrete CXCL1 or IL-8, which attract and activate neutrophils via CXCR1/CXCR2 receptors on the neutrophil surface; ② Adhesive molecules: CD44v6 or MUC1 expressed by CTCs bind to selective receptors on neutrophils, facilitating adhesion between these atypical cells; ③ Immune checkpoint molecules: CD47 expressed by CTCs interacts with SIRPα on neutrophils, inhibiting the phagocytic clearance of tumor cells; ④ NETs: NETs released by neutrophils can directly capture CTCs, forming physical aggregates (16).

CTC-neutrophil aggregates facilitate tumor cells in evading immune-mediated destruction through multiple mechanisms. Firstly, these neutrophils create a physical barrier around the tumor cells, thereby obstructing the interaction between cytotoxic T cells and NK cells (16). Secondly, neutrophils within these aggregates adopt an immunosuppressive phenotype, characterized by elevated expression of Arg-1, which depletes arginine in the microenvironment, consequently inhibiting T cell proliferation and function (10). Additionally, ROS released by neutrophils can induce apoptosis and dysfunction in T cells (50). Notably, the interaction between CTCs and neutrophils is reciprocal. Tumor cells influence the development and function of neutrophils by secreting cytokines such as G-CSF and GM-CSF, which promote their differentiation into immunosuppressive phenotypes (13). This interaction establishes a positive feedback loop, further amplifying immune evasion.

Furthermore, neutrophils play a critical role in enhancing the survival of CTCs through multiple mechanisms. Activated neutrophils release IL-8, which can activate CXCR1/CXCR2 receptors on the surface of CTCs, subsequently inhibiting tumor cell apoptosis via the phosphoinositide 3-kinase (PI3K)/protein kinase B (AKT) signaling pathway (16). Additionally, neutrophils modulate tumor cell signaling pathways by releasing ROS and reactive nitrogen species, inducing a reversible dormant state in tumor cells and increasing their resistance to chemotherapy (58). Within the capillary beds of target organs, CTC-neutrophil aggregates adhere to the vascular endothelium through integrin-mediated adhesion. Neutrophil-derived NEs and MMP-9 degrade endothelial cell junctions and basement membranes, facilitating CTC extravasation (16). NETs are pivotal in this process, as they anchor to the vascular wall, capture circulating CTCs, and compromise the vascular barrier through localized high concentrations of proteases, thereby promoting tumor cell infiltration into tissue parenchyma (58).

#### 3.4.3 Construction PMN in distal organs

PMN is formed after the primary tumor has formed and released remote regulatory signals, and before disseminated tumor cells reach distant organs, thereby creating a conducive milieu for the subsequent colonization by metastatic cells (10). Among the cellular constituents of pre-metastatic lesions, neutrophils play a pivotal role. Tumor cells induce the bone marrow to produce an abundance of neutrophils and facilitate their mobilization into the circulatory system through the secretion of factors such as G-CSF and IL-1β. These neutrophils are subsequently recruited to pre-metastatic sites (10, 13).

A fundamental characteristic of PMN is the establishment of an immunosuppressive milieu, wherein neutrophils assume a pivotal role. TANs polarized towards the N2 phenotype exhibit elevated expression of Arg-1, which catalyzes the degradation of arginine within the microenvironment, thereby inhibiting T cell functionality. TGF-β is abundantly expressed in the PMN, facilitating the polarization of neutrophils towards the N2 phenotype while concurrently suppressing the emergence of the N1 anti-tumor phenotype. This dual mechanism ensures the evasion of tumor cell clearance (10). Neutrophils further contribute to immune suppression by recruiting and modulating other immune cell populations. For instance, CCL2 secreted by neutrophils attracts monocytes, which subsequently differentiate into M2 TAMs within the PMN (58). The interaction between neutrophils and Tregs further consolidates the immunosuppressive network (10).

Neutrophils play a pivotal role in matrix remodeling within PMN. Upon arrival at the pre-metastatic site, neutrophils secrete substantial quantities of proteases, including MMP-9 and NE, which facilitate the degradation of the local ECM. This degradation exposes concealed adhesion sites, thereby providing a scaffold for the attachment of subsequent tumor cells. Additionally, the fragments resulting from matrix degradation can act as chemotactic signals, recruiting further immune cells to the site. Concurrently, neutrophils enhance local vascular permeability in PMN by secreting factors such as VEGF and MMP-9 (10). PMN not only create a conducive environment for the initial colonization of circulating tumor cells but may also play a role in reactivating tumor cells that have disseminated and entered a dormant state. Factors such as chronic inflammation, viral infections, or stress hormones can activate neutrophils within these lesions. The inflammatory mediators released by activated neutrophils, including IL-6 and PE2, may stimulate dormant tumor cells to re-enter the cell cycle (13). NETs are implicated in this process by interacting with integrins located on the surface of tumor cells via laminin. This interaction subsequently activates the focal adhesion kinase (FAK)/extracellular signal-regulated kinases (ERK)/myosin light-chain kinase (MLCK) signaling pathway, thereby inducing the proliferation of dormant cells (58).

### 3.5 B cells

As the central effector cells of humoral immunity, B cells exhibit significant functional plasticity in their biological characteristics during tumor metastasis. It is important to emphasize that B cells are not a monofunctional population. Their antitumor potential includes mechanisms such as the production of tumor-specific antibodies, antigen presentation, and T-cell activation, however, their metastasis-promoting functions are associated with specific B-cell subsets in various tumor contexts (58). For example, IL-10-dependent regulatory B cells (Bregs) in lung metastases of breast cancer (61), IL-10+PD-1+ Bregs and IgA+ plasma cells in hepatocellular carcinoma (62), CXCL13+ B cells in melanoma (63), IL-10+ B cells in liver metastases of colorectal cancer (64), as well as CD40/CD40L- and VEGF-associated B cells in diffuse large B-cell lymphoma and multiple myeloma (65, 66). Therefore, the net effect of B cells on tumor immunity depends on tumor type, site of metastasis, disease stage, and the predominant B-cell subsets.

In some TME, Bregs predominate and exert an immunosuppressive effect. Bregs predominantly secrete inhibitory cytokines such as IL-10, TGF-β, and IL-35, which suppress the activity of effector T cells and natural killer cells while promoting the expansion of regulatory T cells. This process contributes to the establishment of an immunosuppressive microenvironment, thereby creating favorable conditions for tumor metastasis (19). Studies have demonstrated that in various tumor models, Breg infiltration is positively correlated with metastatic burden. For instance, in a murine breast cancer lung metastasis model, tumor-infiltrating Bregs inhibit the cytotoxic function of CD8+ T cells in an IL-10-dependent manner, thereby promoting lung metastasis formation (61). In human hepatocellular carcinoma, IL-10+PD-1+ Bregs have been shown to suppress CD8+ T cell responses, which is associated with tumor metastasis and poor prognosis (62). Notably, Bregs can also indirectly inhibit anti-tumor immunity by inducing Tregs. Studies have demonstrated that the CD25+B220+CD19+B cell subset is capable of interacting with CD4+ T cells, secreting TGF-β, and inducing the differentiation of CD4+ T cells into FoxP3+ Tregs, thereby facilitating pulmonary metastasis (67). This synergistic interaction between Bregs and Tregs establishes a positive feedback loop that enhances immune suppression, further intensifying immune evasion within the metastatic microenvironment.

B cells have the potential to facilitate metastasis by inhibiting antibody-dependent cell-mediated cytotoxicity (ADCC). While certain antibody subclasses are capable of eliminating tumor cells via ADCC and complement-dependent cytotoxicity (CDC) mechanisms, specific antibodies, particularly certain IgG subclasses and IgA, can instead enhance tumor metastasis through Fc receptor-dependent pathways (68). In a murine model of squamous cell carcinoma, IgG produced by tumor-associated B cells engages the activated Fcγ receptors on myeloid cells. This interaction promotes the recruitment and activation of myeloid-derived suppressor cells, which subsequently suppress the CD8+ T cell response, thereby accelerating tumor progression and metastasis (69). The tumor-promoting effect was abrogated in mice deficient in activated Fc γ R, thereby confirming the critical role of the antibody Fc γ R axis in facilitating metastasis. IgA+ plasma cells represent another subset of B cells implicated in the promotion of metastasis. In hepatocellular carcinoma, IgA+ plasma cells exhibit high expression levels of IL-10 and PD-L1, which contribute to tumor immune evasion and metastasis by inhibiting the proliferation and cytotoxic activity of CD8+ T cells (70). Collectively, these mechanisms underscore the pivotal role of B cells as regulators of tumor immune escape.

B cells play a pivotal role in the establishment of the PMN through the secretion of angiogenic factors and matrix remodeling enzymes. Upon activation, B cells are capable of producing factors such as VEGF, bFGF, and MMP-9. These factors stimulate endothelial cell proliferation and migration, promote angiogenesis, and enhance vascular permeability, thereby facilitating the hematogenous dissemination of tumor cells (10). Studies have demonstrated that, in response to factors secreted by primary tumors, B cells within lung tissue can become activated and produce VEGF, which increases local vascular permeability, promotes the recruitment of myeloid-derived suppressor cells, and contributes to the formation of a pre-metastatic microenvironment that is conducive to tumor cell colonization (71). B cells play a crucial role in modulating the invasive capacity of tumor cells via lymphotoxin signaling. In prostate cancer models, lymphotoxins produced by tumor-infiltrating B cells activate the NF-κB and STAT3 signaling pathways within tumor cells, thereby enhancing their survival capabilities and facilitating the transformation to a mesenchymal phenotype, ultimately promoting metastasis (55). These findings suggest that the mechanisms underlying B cell-mediated metastasis promotion differ across tumor types and that the specific role of B cells is contingent upon the intrinsic characteristics of the tumor cells and the regulatory signals within the microenvironment.

### 3.6 NK cells

Recent studies suggest that the role of the innate immune system in tumor metastasis should be expanded to include the entire family of innate lymphoid cells (ILCs). ILCs include NK cells, ILC1, ILC2, ILC3, and lymphoid tissue-induced cells (LTi). They are regulated by complex molecular signaling networks in the TME, including cytokines (IL-12/15/18/23, TGF-β), chemokines (CXCL9/10/11-CXCR3, CX3CR1, CCL2/CCR2), metabolic hypoxia (lactate, ROS, ARG1, IDO), and immune checkpoints (PD-1/PD-L1), and shape the immune microenvironment at different stages of the metastasis cascade (45). It is worth noting that the development and subpopulation balance of ILC3 are also finely regulated by the nuclear receptor Nur77 and its endogenous ligand 12R-HETE, which drive NKp46^+^ ILC3 differentiation via T-bet/Impdh1 and enhance IFN-γ production (72). Although this study focused on anti-infectious immunity in the gut of neonatal mice, it suggests that ILC3 subset balance is regulated by an endogenous metabolite–nuclear receptor axis and a similar signaling logic in the TME may be involved in regulating ILC3 pro-tumor polarization, warranting further exploration. These signals originate from tumor cells as well as TAMs, MDSCs, N2-type neutrophils, Bregs, DCs, and stromal cells, forming a dynamic “molecular signaling–ILC–immune microenvironment” axis (45, 73). The remarkable plasticity of ILCs makes them a “double-edged sword” in tumor immunity, they can drive antitumor responses, but under specific conditions, they can also promote tumor progression and metastasis (45, 73).

NK cells, traditionally recognized as pivotal effector cells in the suppression of metastasis, serve as the primary defenders within innate immunity. However, recent studies (13, 74) highlight that within the intricate signaling networks of the TME, NK cells can both exert cytotoxic effects to eliminate metastatic tumor cells and undergo reprogramming into tumor-associated NK cells (TANKs) that suppress CD8^+^ T cell immunity. Furthermore, they may indirectly facilitate tumor metastasis through various mechanisms.

TANKs are capable of constraining antitumor immunity through various mechanisms, including competition with CD8^+^ T cells for IL-2 and IFN-1, interference with the differentiation and function of effector T cells, and suppression of adaptive immune responses via immune editing and regulation of dendritic cell function (13). Two studies have provided reliable mechanistic evidence supporting the immunosuppressive role of TANKs. Pozniak et al. identified that cytotoxic NK cells are recruited to tumor sites via the CX3CR1 receptor in immunosuppressive melanoma, forming a physical barrier that impedes CD8^+^ T cell infiltration, thereby diminishing the effectiveness of anti-PD-1 therapy (74). Similarly, Song et al. demonstrated that TANKs compete with CD8^+^ T cells for IL-2 and type I interferons, disrupting the differentiation of effector T cells and reducing their sensitivity to immune checkpoint blockade (74). These findings fundamentally challenge the traditional notion of NK cells as constantly antitumor and position them within a more complex immune regulatory network (13). However, it must be clearly acknowledged that the aforementioned studies focused on specific tumor models, specific immunotherapy contexts, and specific interaction mechanisms. Therefore, it cannot be inferred from these findings that TANKs are universally present and play a dominant role in all tumor types, all metastatic sites, or all treatment settings. There is currently a lack of independent validation across cancer types, metastasis stages, laboratories, microenvironment contexts, and prospective clinical samples. Therefore, TANKs represent an emerging functional state or mechanistic hypothesis of NK cells identified within specific TME and immunotherapy contexts, whether they constitute a broadly applicable paradigm requires confirmation through further independent studies.

### 3.7 DCs

DCs, recognized as the most potent specialized antigen-presenting cells within the body, function as a critical interface between innate and adaptive immunity and have traditionally been regarded as pivotal in the initiation and maintenance of anti-tumor immune responses. Nevertheless, research over the past decade has increasingly demonstrated that DCs are subject to functional reprogramming under the intricate regulatory signaling networks of the TME. This reprogramming can shift their role from being central agents of immune activation to becoming accomplices that facilitate tumor progression and metastasis (12).

The generation of immunosuppressive DCs subsets constitutes a fundamental mechanism facilitating tumor metastasis. Studies have shown that mature dendritic cells enriched in immunoregulatory molecules (mregDCs) are closely associated with naive T cells and B cells while being enriched in immunoregulatory molecules, and their abundance can predict progression-free survival following treatment with immune checkpoint inhibitors. At the same time, mregDCs also highly express immunosuppressive molecules (such as PD-L1, IDO1) and chemokine receptor CCR7, while lowly expressing co-stimulatory molecules (CD80, CD86). They are induced by immunosuppressive factors (e.g., IL-10, TGF-β), metabolic stress, and hypoxia in the TME, which can inhibit effector T cell function and promote Tregs expansion, serving as a key mechanism for tumor immune evasion (75, 76). However, it is important to note that mregDCs are not a monofunctional population, whether immune activation or immune suppression predominates is not fixed but is determined by a combination of subpopulation classification, tissue localization, TME signaling networks, and interactions among immune cells (75).

Furthermore, plasmacytoid dendritic cells (pDCs) and specific subpopulations of myeloid DCs have been shown to induce the production of Tregs through the secretion of high levels of immunosuppressive cytokines, such as IL-10, thereby playing a critical role in tumor-mediated immune suppression. Studies have demonstrated that in cervical cancer, cervical epithelial cells exploit their aberrant sialylation surface structures to transform conventional dendritic cells into a phenotype characterized by low immunogenicity and high immune tolerance. DCs expressing Siglec-10 further inhibit the function of adaptive T cells via galectin-9 signaling, thereby reinforcing the immunosuppressive microenvironment. Disruption of Siglec-10 signaling can restore the dendritic cell-mediated tumoricidal response and enhance the sensitivity of adaptive T cells to PD-1 inhibition. This finding identifies Siglec-10 as a significant inhibitory checkpoint on dendritic cells, offering a novel target for the immunotherapy of cervical cancer (10).

The interaction between DCs and Tregs serves as the central axis of the immunosuppressive network. Studies have demonstrated that, in various cancer models, there is a significant increase in CXCR3^+^ Tregs within tumors compared to lymphoid tissues, with these Tregs preferentially interacting with BATF3^+^ DC1s that secrete CXCL9. Genetic ablation of CXCR3 in Tregs can disrupt the interaction between DC1s and Tregs, thereby enhancing the interaction between DCs and CD8^+^ T cells. Mechanistic investigations have revealed that the absence of CXCR3 in Tregs augments DC1-mediated tumor antigen-specific cross-presentation, increases the priming and reactivation of CD8^+^ T cells, and ultimately inhibits tumor progression (77). It is noteworthy that certain DC activation strategies may inadvertently promote Treg recruitment while simultaneously enhancing anti-tumor immunity. In the context of pancreatic ductal adenocarcinoma (PDAC), although the combination of Flt3L and CD40 agonists can restore the number and function of conventional dendritic cells (cDCs) and elicit type I immune responses driven by cDCs, the activation of cDC2 paradoxically increases Treg numbers and attenuates the immune response (58). The findings elucidate the intricate nature of the DCs and Tregs axis in the regulation of anti-tumor immunity. They indicate the necessity for a synergistic approach in targeting Tregs recruitment signaling pathways to enhance therapeutic efficacy, while concurrently activating the anti-tumor functions of DCs.

In addition, dendritic cell-derived exosomes (DC-EVs) exhibit a dual role in tumorigenesis. They have the capacity to interact with other immune cells and activate anti-tumor immunity through the expression of surface molecules such as MHC and co-stimulatory molecules. Conversely, they can be reprogrammed by the TME to facilitate inflammatory responses, angiogenesis, and immune evasion (12). DC-EVs modulate T cell activation by transporting molecules such as miR-155, convey Wnt/β-catenin signaling to enhance tumor metastasis, and influence the VEGF pathway to impact angiogenesis (12). This bidirectional regulatory capability positions DC-EVs as a double-edged sword in tumor immunotherapy, offering both significant potential and necessitating careful consideration.

Within the intricate regulatory framework of the TME, DCs play a dual role: they can present antigens to activate anti-tumor immunity or be reprogrammed into immunosuppressive phenotypes, such as mregDCs and Siglec-10^+^ DCs, which facilitate tumor immune evasion and metastasis. This reprogramming occurs through mechanisms including the DC-Treg axis, chemokine signaling pathways, and intercellular communication mediated by extracellular vesicles.

It is worth noting that the mechanisms by which immune cells promote metastasis not only involve functional reprogramming of immune cells but are also closely related to tumor cell death pathways, abnormalities in intrinsic tumor cell signaling, glycan-mediated immune evasion, and epigenetic regulation of antitumor immunity. Studies have shown that various cell death pathways—including apoptosis, necrotic apoptosis, pyroptosis, ferroptosis, and immunogenic cell death—can release damage-associated signaling molecules, inflammatory factors, and metabolites, which in turn influence DC maturation, antigen cross-presentation, CD8^+^ T-cell activation and exhaustion, and reshape the pre-metastatic microenvironment (78–80). Concurrently, epigenetic mechanisms such as DNA methylation, histone modifications, chromatin remodeling, and non-coding RNAs can regulate the expression of tumor antigens, MHC class I/II molecules, PD-L1, chemokines, and immunosuppressive molecules, thereby influencing the functional state of T cells, NK cells, DCs, and myeloid cells (81, 82). In addition, abnormal signal transduction in tumor cells and surface glycan remodeling are key drivers of immune evasion and the shaping of the metastatic microenvironment. On the one hand, tumor cells can, through the sustained activation of signaling pathways such as PI3K/AKT, MAPK/ERK, JAK/STAT, Wnt/β-catenin, NF-κB, and TGF-β/SMAD, upregulate immune checkpoint molecules and immunosuppressive cytokines/chemokines, alter MHC-I/II antigen processing and presentation, weaken the cytotoxic function of CD8^+^ T cells and NK cells, and promote the recruitment and polarization of suppressive cells (e.g., Tregs, MDSCs, and TAMs) in the PMN (9, 83). On the other hand, glycan alterations on the tumor cell surface—such as increased sialylation, fucosylation, N-glycosylation branching, and abnormalities in mucin-type O-glycosylation—can interact with Siglec family receptors (e.g., Siglec-7/9/10/15), galectins (e.g., Galectin-9), and selectins, directly inhibiting NK cell and T cell activation, inducing the differentiation of tolerant DCs and Tregs, or masking antigenic epitopes, thereby forming “glycan immune checkpoints” (84, 85). These intrinsic signaling pathways in tumor cells and glycan-mediated immune evasion mechanisms are coupled with the aforementioned immune cell-mediated mechanisms that promote metastasis, further expanding our understanding of the immunological mechanisms underlying tumor metastasis.

## 4 Conclusions

Tumor metastasis is not driven solely by the autonomous actions of tumor cells but stems from dynamic interactions between tumor cells and the immune system. Today, immune cells are no longer viewed as mere passive bystanders or responders but are widely recognized as active participants in the metastasis process (**Figure 1**, **Table 1**). Under the influence of tumor-derived signals, such as cytokines, chemokines, metabolites, and extracellular vesicles, these immune cells undergo phenotypic shifts, transitioning from an antitumor state to one that promotes metastasis. They facilitate tumor cell metastasis through various mechanisms, including promoting tumor cell invasion, aiding tumor cell survival in the circulatory system, forming PMNs, and establishing an immunosuppressive microenvironment. This represents one of the key mechanisms of tumor metastasis. However, numerous questions remain to be addressed, such as understanding the regulatory networks governing immune cell dynamics during metastasis, differences in the immune microenvironment across various organs, and the influence of host factors such as immunosenescence and obesity. Future research should utilize techniques such as single-cell sequencing, spatial transcriptomics, and *in vivo* imaging to elucidate the heterogeneity and interactions of immune cells at different stages of metastasis and across various organs, thereby advancing our understanding of the mechanisms by which immune cells promote metastasis.

**Figure 1:**
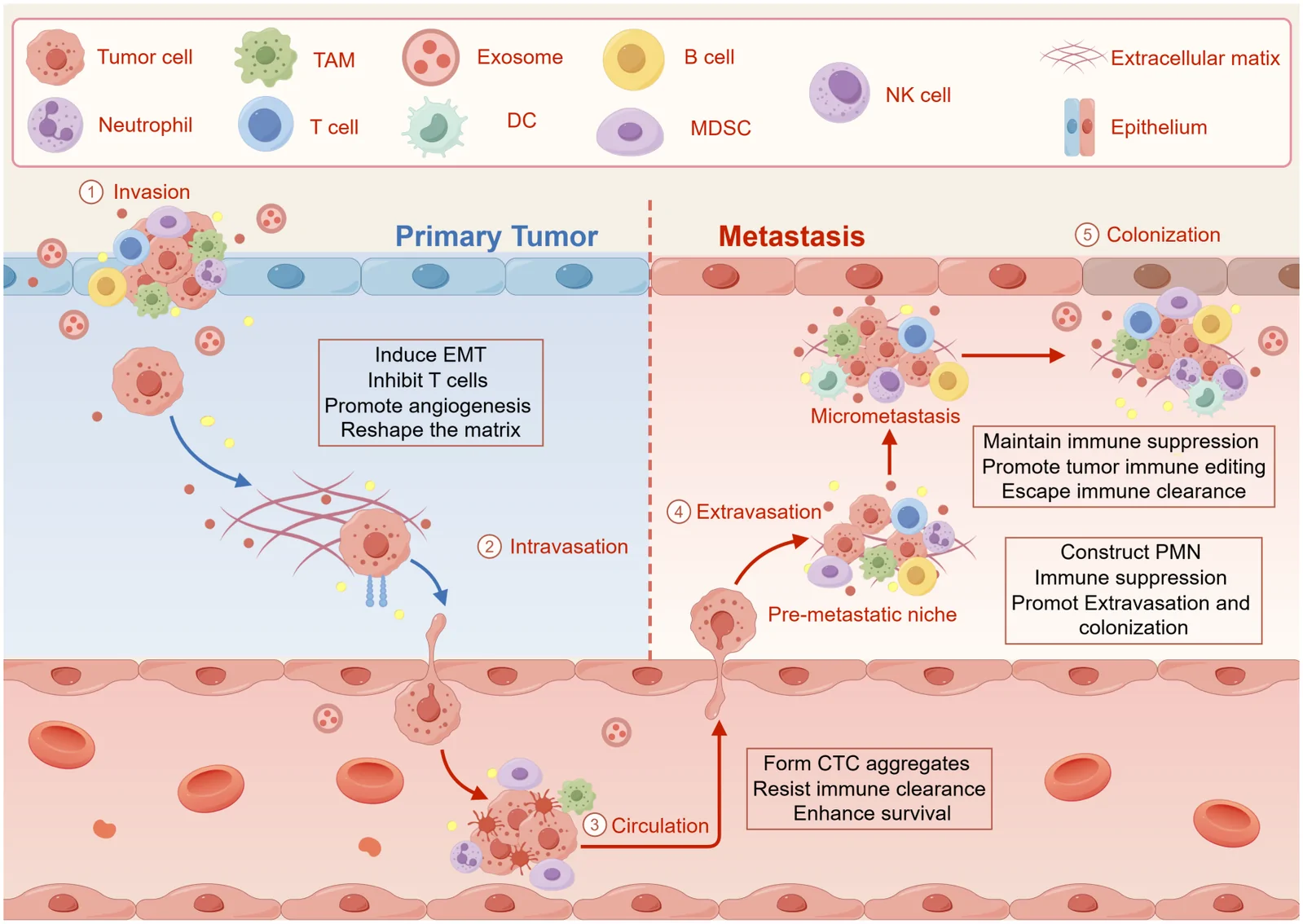
The metastasis-promoting role of immune cells in tumor metastasis. The immune regulation of tumor metastasis is a complex network system involving multiple cell types, multiple signaling pathways, and various temporal and spatial stages. T-cell dysfunction, functional remodeling of TAMs, immunosuppression by MDSCs, pro-metastatic polarization of neutrophils, immunomodulation by Bregs, functional reprogramming of NK cells, and the immunosuppressive transformation of DCs collectively constitute the immunological basis for successful tumor metastasis. These immune cells do not act in isolation; rather, they form an immunosuppressive ecosystem that mutually reinforces and synergistically amplifies each other through various mechanisms, thereby supporting all stages of tumor metastasis. (The Figure completed by figdraw.com).

**Table 1:** Summary of immune cell subsets, metastatic stages, experimental evidence, and key mediators/mechanisms in tumor metastatic.

Immune cell subset	Metastatic stage	Representative tumor types	Key mediators/mechanisms	Experimental model system	Evidence level
CD8^+^ exhausted T cells	Primary tumor, circulation, PMN	Colorectal cancer, melanoma	PD-1, TIM-3, metabolic stress, mitochondrial transfer	Mouse models, patient specimens, scRNA-seq	Established
Tregs	Primary tumor, lymph node, PMN	Breast cancer, HCC	IL-2 consumption, IL-10, TGF-β, CCL22/CCL28	Mouse models, patient specimens	Established
TAMs	All stages	Breast cancer, colorectal cancer, glioma	TGF-β, VEGF, MMPs, PD-L1, MHC-I degradation	Mouse models, spatial transcriptomics, scRNA-seq	Established
PMN-MDSCs	PMN, circulation	Head/neck cancer, breast cancer	ARG1, ROS, NETs, CXCL2/8	Mouse models, patient blood	Established
M-MDSCs	Primary tumor, circulation	Glioblastoma	CCL2/CCR2, IL-10, TGF-β	Mouse models, scRNA-seq	Emerging
TANs	Invasion, circulation, PMN	Gastric cancer, breast cancer, HCC	NE, MMP-9, NETs, ARG1, S100A8/A9	Mouse models, scRNA-seq, spatial	Emerging
Bregs	PMN, metastatic niche	Breast cancer, HCC, melanoma	IL-10, TGF-β, IgG-FcγR, VEGF	Mouse models, patient specimens	Emerging
TANKs	Primary tumor, metastatic niche	Melanoma	IL-2/IFN competition, CX3CR1 barrier	Mouse models, patient specimens	Emerging
DCs	Lymph node, PMN	Melanoma, NSCLC, cervical cancer	IL-10, galectin-9, C5a/C5aR1, DC-EVs	Mouse models, patient specimens	Emerging
